# Hospitals and the Spirit Duties

**Published:** 1915-07-24

**Authors:** F. A. Hocking

**Affiliations:** London Hospit 1, Whitechapel Road, E.


### Hospitals and the Spirit Tax.

*To the Editor of* The Hospital.

### Hospitals and the Spirit Duties.

*To the Editor of* The Hospital.

Sir,—On July 11 I wrote to the Editor of the *Pharmaceutical Journal* embodying the views outlined below, but my letter does not appear in the current issue, although room is found for another letter (written over a *nom de plume*), presumably because the views contained therein are more acceptable to the Pharmaceutical Society.

*Apropos* of your leader last week I would urge all interested in hospital work to endeavour to enlist the support of as many M.P.s throughout the constituencies as possible for Mr. Bridgeman's clause.—Yours faithfully, F. A. Hocking.

London Hospit l, Whitechapel Road, E.,

July 11, 1915.

[Below is the text of the letter referred to.—Ed.]

*The Editor of the "Pharmaceutical Journal."*

Dear Sir,—A curious claim put forward by the editor of the *Pharmaceutical Journal* on behalf of the Pharmaceutical Society is well worth the serious attention of all interested in hospital work.

In the recent discussion on "Hospitals and Spirit Duty" I suggested that if a committee were appointed to consider the question, provision should be found for the representation thereon of hospital views.

The comment of the editor of the *Pharmaceutical Journal* is to the effect that hospital opinions on this subject are sufficiently and efficiently represented by the president and secretary of the Pharmaceutical Society because of their past experience as hospital pharmacists, the former as pharmaceutist to St. Thomas's Hospital and the latter as a dispenser at University College Hospital. This claim can scarcely pass unchallenged, for two reasons:

(i.) Both the president and secretary have long ago ceased their connection with hospital pharmacy, and are, therefore, out of touch with later developments.

(ii.) It is impossible for one person to represent with justice, at one and the same time, two parties whose views are certainly not in agreement, but may even be in direct conflict.

One's faith in such presentation of hospital views on the duty-free spirit question is not rendered any more robust by the hostility displayed by the parliamentary secretary of the Pharmaceutical Society towards Mr. Bridgeman's proposals, hostility based on a rudimentary and distorted knowledge of hospital pharmacy.

Yours faithfully,
F. A. Hocking.

